# Locally biosynthesized gibberellins in *Populus* stems are involved in the regulation of wood development

**DOI:** 10.48130/forres-0025-0005

**Published:** 2025-02-27

**Authors:** Huili Su, Chunyang Wan, Xiaokang Fu, Jian Hu, Yuanxun Tao, Keming Luo, Changzheng Xu

**Affiliations:** Chongqing Key Laboratory of Tree Germplasm Innovation and Utilization, Integrative Science Center of Germplasm Creation, School of Life Sciences, Southwest University, Chongqing, China

**Keywords:** Gibberellins, *Populus*, Secondary growth, Cambium, Wood forma

## Abstract

Gibberellins (GAs) are a class of hormonal regulators, which influence various developmental processes in the life cycle of plants. In woody species, bioactive GAs, whose precursors are synthesized by the action of terpene cyclases and carried in the phloem by long-distance translocation from aerial shoot apex and leaves, are known to be a mobile signal to modulate stem growth. However, little is known about the existence and role of local GA synthesis in stems. Here we provide multiple lines of evidence suggesting the presence of local *de novo* GA biosynthesis in poplar stems and assess its role in wood development. First, the application of a GA biosynthetic inhibitor to decapitated poplar led to a significant reduction in local GA accumulation in the stem. Second, the correlated expression patterns of GA biosynthetic genes across radial tissues showed the existence of local GA production in the stem. Third, bioactive GA assays in transgenic poplar lines expressing Arabidopsis *CPS*, which encodes the first enzyme in the GA biosynthetic pathway, further confirmed the occurrence of local GA biosynthesis in the bark and cambial zones, but not in the xylem. Finally, modified local GA biosynthesis in the stem revealed its positive effects on secondary growth during wood formation. Taken together, our results demonstrate the existence of local *de novo* biosynthesis of GAs in poplar stems that contributes to the regulation of wood development via stimulating cambial activity.

## Introduction

Gibberellins (GAs), a group of tetracyclic diterpenoids, function as endogenous hormonal signals that play a crucial role in regulating both vegetative and reproductive development in plants^[1,2]^. The role of GAs in promoting plant growth was first demonstrated through the excessive stem elongation phenotype in rice, induced by metabolites secreted by a pathogenic fungus, which were later identified as GAs^[3]^. Genetic and biochemical studies in Arabidopsis and cereal species have elucidated the dynamic and coordinated regulation of GA homeostasis and signal transduction during stem elongation^[1]^. Unlike herbaceous plants, perennial trees undergo secondary growth in their stems, leading to radial thickening and wood formation alongside longitudinal elongation^[4]^. GAs play a critical role in wood development, particularly in the differentiation of xylem fibers^[5,6]^.

Secondary growth in tree stems arises from continuous cell proliferation in the vascular cambium, a lateral meristem^[7]^. Cambial cells undergo a series of processes during wood formation, including xylem cell specification, expansion, secondary wall deposition, and apoptosis^[8]^. GA metabolism and signaling pathways are evolutionarily conserved across woody species^[2,9]^. Exogenous application of GA to decapitated trees stimulates multiple cellular processes involved in secondary stem growth^[10,11]^. For instance, the elongation of xylem fibers is influenced by the application of GA biosynthesis inhibitors to woody stems^[12]^. Increased GA levels, achieved through overexpression of *GA20 oxidase* (*GA20ox*), a key enzyme in GA biosynthesis, lead to significant changes in xylogenesis in poplar^[5,13]^. Transcriptome profiling of these transgenic plants reveals GA-mediated transcriptional regulation of genes involved in various cellular processes during xylem development^[13]^. In contrast, altered expression of two *GA2 oxidase* genes results in varying wood formation characteristics in poplar^[14]^. Constitutive and xylem-specific expression of *GID1* paralogs, encoding GA receptors, produced xylogenesis phenotypes similar to *AtGA20ox* overexpressors, albeit with distinct xylem fiber traits compared to *35S:AtGA20ox1* transgenic lines^[15]^. These divergent roles of GAs in xylogenesis and fiber development during secondary growth highlight the necessity of tissue-specific signaling pathways^[15]^, underscoring the complexity of GA-mediated wood formation in trees.

The spatiotemporal distribution of GAs is tightly regulated by the stage- and cell-type-specific expression of their biosynthetic and catabolic enzymes^[2,9]^. Due to their mobility, the transport of bioactive GAs and their intermediates significantly influences their accumulation patterns^[16,17]^. Studies have shown that early and late steps in GA biosynthesis occur in distinct locations during Arabidopsis seed germination, suggesting intercellular transport of intermediates for bioactive GA production^[18]^. GAs synthesized in the stamen not only act locally in Arabidopsis but also move to neighboring petals for non-autonomous functions^[19]^. Micrografting and biochemical approaches further revealed long-distance transport of bioactive GAs and intermediates from synthesis sites to tissues or organs requiring GA activity^[20,21]^. Thus, both local biosynthesis and intercellular transport cooperatively regulate GA distribution and function.

Shoot-derived GAs have been characterized as mobile signals in stem development in both herbaceous and woody plants^[22]^. In Arabidopsis hypocotyls, GAs act as mobile signals derived from the shoot, directly inducing xylogenesis upon flowering^[23]^. Similarly, mobile GAs synthesized in leaves influence various aspects of secondary growth when transported to stems in tobacco^[24]^. GA_12_, an inactive precursor, is the primary form of GA transported over long distances along the vasculature in Arabidopsis^[21]^. In tree stems, bioactive GAs (primarily GA_1_ and GA_4_) peak in the developing xylem^[25,26]^. Exogenous GA application at the decapitation site of poplar trees increases GA levels in secondary tissues of the stem, suggesting phloem-directed downward translocation of GAs or their precursors from the shoot apex to wood-forming tissues^[11]^. Notably, dual concentration peaks of GA precursors (GA_9_ and GA_20_) are observed, with one peak in the expanding xylem overlapping with bioactive GAs and another in the bark, including the phloem and cambial zone^[25]^. Additionally, spatially resolved analyses of GA biosynthetic genes reveal high expression of *CPS1*, the first enzyme in *de novo* GA biosynthesis, in the bark tissue^[25]^. These findings suggest the possibility of *de novo* GA synthesis in tree stems, although direct evidence remains lacking.

In this study, we identified local *de novo* GA biosynthesis in the bark and cambium tissues of *Populus tomentosa*. Evidence from the exogenous application of a GA biosynthesis inhibitor, modulation of endogenous GA levels, and spatiotemporal expression analysis of GA biosynthetic genes demonstrate that locally synthesized GAs promote secondary growth by stimulating cambial activity. Our findings provide new insights into the coordinated production of GAs and their role in wood formation in trees.

## Materials and methods

### Plant material and growth conditions

*Populus tomentosa* Carr. (Clone 741) was micropropagated *in vitro* on Woody Plant Medium (WPM). Genetic transformation of *P. tomentosa* was conducted using *Agrobacterium*-mediated leaf disc infection, as described in a previous study^[27]^, with selection on hygromycin (9 mg/L). Putative transformed plants were confirmed by PCR genotyping using gene-specific primers (Supplementary Table S1). The transformed plants were subsequently cultivated in a greenhouse at 25 °C under a 16-h light/8-h dark photoperiod, with supplemental light at 5,000 lux and 60% relative humidity.

### RNA extract and quantitative real-time PCR

Total RNA was extracted from the stems of 2-month-old *Populus* plants using the Plant RNeasy Mini Kit (Qiagen). cDNA synthesis was performed using the PrimeScript™ RT Reagent Kit with random hexamer primers and 200 ng of total RNA. Quantitative real-time polymerase chain reaction (RT-qPCR) was carried out using SYBR Premix Ex Taq™ (Takara, Dalian, China) on a TP800 Real-Time PCR system (Takara, Japan). The poplar ubiquitin gene served as the reference for normalization of the expression data. Primer sequences used in RT-qPCR are provided in Supplementary Table S1.

### Gene cloning and vector construction

The full-length coding sequence of *AtCPS* was amplified from the cDNA of *Arabidopsis thaliana* using a gene-specific primer (Supplementary Table S1). The PCR products were then ligated into the pCXSN vector via TA-cloning^[28]^, under the control of the *CaMV 35S* promoter. The *pro35S-AtCPS* fragments were subsequently inserted into a modified *pCAMBIA1305* vector, in which the hygromycin-resistant gene was replaced with the kanamycin-resistant gene to facilitate positive selection of transgenic plants through homologous recombination. Additionally, 2-kb upstream promoter regions of the *ANT*^[26]^ and *LMX5*^[15]^ genes were amplified from the genomic DNA of *Populus tomentosa* using gene-specific primers (Supplementary Table S1) and integrated into the modified *pCAMBIA1305* vector by homologous recombination to drive *AtCPS* expression.

### Phylogenetic analysis

Multiple sequence alignments were performed using DNAMAN 8 (Lynnon Biosoft, San Ramon, USA), and phylogenetic tree construction was carried out with MEGA 6.06^[29]^.

### Plant transformation

*P. tomentosa* was stably transformed using Agrobacterium-mediated leaf disk infiltration, as previously described^[27]^. PCR amplification with primers specific to hygromycin/kanamycin resistance genes (Supplementary Table S1) was performed to confirm the integration of the transgene into the genome of the transgenic plants. Both transgenic and wild-type (WT) poplar plants were propagated via *in vitro* microcutting. Shoot segments (3–4 cm in length with 2–3 leaves) were excised from sterilized seedlings and cultured on WPM medium for 4 weeks at 25 °C under a 16-h light (5,000 lux) and 8-h dark cycle.

### Decapitation and PAC treatments

For decapitation treatment, 2-week-old sterilized poplar seedlings, grown on WPM, had their top buds and side branches removed to inhibit the production of bioactive gibberellins (GA). These plants were then continuously cultivated at 25 °C under a 16-h light cycle at 5,000 lx, followed by 8 h of darkness. For Paclobutrazol (PAC) treatment, decapitated poplar plants were cultured on WPM supplemented with 10 μM PAC.

### Measurement of GA_4 _content

Four-week-old poplar plants cultivated in WPM were used for GA_4_ content analysis. The second to eighth stem internodes were harvested and immediately frozen in liquid nitrogen at −70 °C. For GA_4_ quantification, the frozen plant material was ground in liquid nitrogen. GA_4_ concentrations were determined using a GC/MS-SRM instrument (JMS-MStation 700, JEOL).

### Cross-sectioning and histological staining

The seventh internode (counted from the apex) of 3-month-old poplar plants was sectioned using a razor blade and subsequently stained with 0.05% (w/v) toluidine blue for 5 min. The cross-sections were observed and captured under a Zeiss microscope. ImageJ software (https://imagej.nih.gov/ij/) was used to analyze the images and quantify the morphological parameters of cambium and xylem cells.

### Statistical analysis

All quantitative data were statistically analyzed using Student's t-test and one-way analysis of variance (ANOVA). For post-hoc comparisons, Dunnett's or Tukey's test was employed to identify significant differences between pairs of samples (*: *p* < 0.05; **: *p* < 0.01; ***: *p* < 0.001).

## Results

### Local *de novo* biosynthesis of bioactive GAs exists in poplar stems

**Figure 1:**
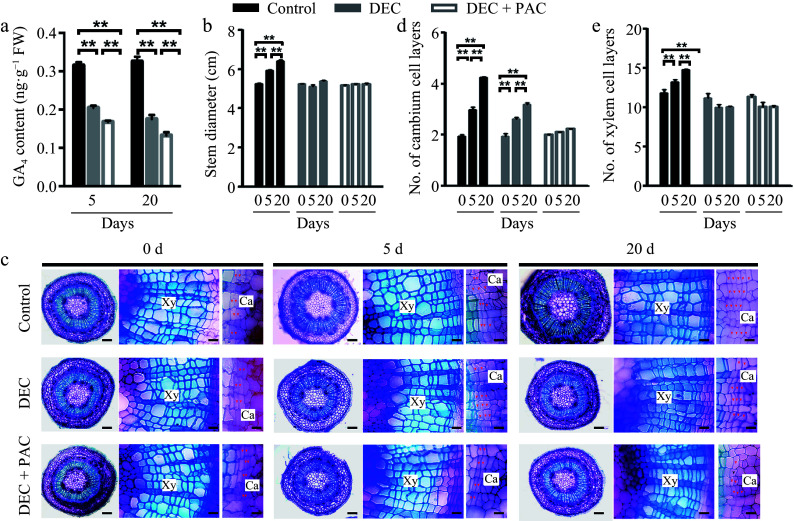
Determination of local *de novo* synthesis of bioactive GAs in poplar stems. (a) GA_4_ levels in poplar stems were measured after 5 or 20 d of decapitation and/or paclobutrazol (PAC) treatments using GC-MS. Error bars represent ± standard deviation (SD). Asterisks denote statistically significant differences between control and decapitation and/or PAC treatments (one-way ANOVA followed by Tukey's test for pairwise comparisons; **, *p* < 0.01; n = 3). (b) Stem diameter was measured after 5 or 20 d of decapitation and/or PAC treatments. (c) Cross-sections of wild-type (WT) poplar plants subjected to decapitation and PAC treatments, grown in WPM medium for 5 and 20 d, respectively. Red arrows indicate cambium cells. Scale bars: left, 250 μm; middle and right, 50 μm. (d), (e) Quantification of cambium and xylem cell layers in poplar stems following decapitation and/or PAC treatments. Error bars represent ± SD. Asterisks indicate significant differences in cambium and xylem cell layers between decapitation and PAC treatments (one-way ANOVA followed by Tukey's test for pairwise comparisons; **, *p* < 0.01; n = 4). DEC, decapitation; PAC, 10 μM paclobutrazol.

Gibberellins (GAs) translocated from the shoot apex and leaves have been demonstrated to play a crucial role in stem growth^[11,23,24]^. To determine whether GAs are locally synthesized in stem tissues, we developed a cultivation system using decapitated poplar plants, which lacked the shoot apex and leaves, thereby eliminating any translocated GAs. Quantitative assays revealed that the endogenous levels of bioactive GA_4_ in poplar stems were significantly reduced 5 and 20 d after decapitation (DEC) compared to the undecapitated control (Fig. 1a). When paclobutrazol (PAC), a chemical inhibitor that interferes with the conversion of kaurene to kaurenol during GA biosynthesis, was applied to the decapitated plants, GA accumulation in the stems was further diminished due to the removal of the source of bioactive GAs from the shoot apex and leaves (Fig. 1a). Furthermore, the stem diameter of control plants increased as the plants grew, whereas the increase in stem diameter was almost completely inhibited in the decapitated plants treated with PAC (Fig. 1b).

To investigate the role of locally synthesized bioactive GAs in wood development, we stained cross-sections of poplar stems with toluidine blue following decapitation and PAC treatments and observed them under a light microscope. As shown in Fig. 1c, secondary vascular development in poplar stems was suppressed following decapitation and PAC treatment. Quantitative measurements revealed that the number of cambial cell layers decreased by 30% after 20 d of decapitation compared to the control (Fig. 1d). When treated with PAC, cambial cell division was completely inhibited in the decapitated stems (Fig. 1d). In contrast, the number of xylem cell layers did not increase following decapitation or PAC treatment (Fig. 1e). These results suggest that bioactive GAs in poplar stems regulate cell division and differentiation in the cambial zone, with the absence of local GA synthesis leading to reduced cambial cell division but not affecting cell differentiation.

To verify changes in GA concentration in the poplar stems following decapitation, we measured the expression levels of two GA-responsive genes, *GASA4* and *EXPA5*^[30]^. RT-qPCR analysis revealed that *GASA4* and *EXPA5* were induced by GA_4_ treatment after 6 h (Supplementary Fig. S1a). Their transcript abundance was significantly reduced 5 d after decapitation and further decreased following PAC treatment (Supplementary Fig. S1b). These results indicate the presence of local *de novo* GA biosynthesis in poplar stems.

### Expression patterns of GA biosynthetic genes in poplar stems

Previous studies have demonstrated that the gibberellin (GA) biosynthetic pathway involves a series of catalytic enzymes, including *ent*-copalyl diphosphate (*ent*-CDP) synthase (CPS), *ent*-kaurene synthase (KS), *ent*-kaurene oxidase (KO), *ent*-kaurenoic acid oxidase (KAO), GA 20-oxidase (GA20ox), and GA 3-oxidase (GA3ox) in Arabidopsis^[31]^. GA biosynthesis is generally divided into three stages, corresponding to three distinct subcellular compartments: plastids, the endomembrane system, and the cytosol (Supplementary Fig. S2a)^[31,32]^. To assess whether a complete *de novo* GA biosynthesis pathway exists in the stems of *Populus* species, we analyzed the expression patterns of genes encoding GA biosynthetic enzymes in poplar stem tissues. Based on evolutionary homology, we predicted and identified all components of the GA biosynthesis pathway in *Populus trichocarpa* (Supplementary Fig. S2a). Gene expression patterns were evaluated across various poplar tissues using microarray data from the EFPop platform (http://bar.utoronto.ca/efppop/cgi-bin/efpWeb.cgi). We found that at least one gene encoding a GA biosynthetic enzyme was highly expressed in the stem (Supplementary Fig. S2b).

To further investigate the expression profiles of these highly expressed genes in the stem, we evaluated their transcript levels in wood-forming tissues of poplar using high-resolution RNA sequencing data^[33]^. *PtrCPS1* and *PtrKS*, as well as *PtrKO* and *PtrKAO*, exhibited similar expression patterns, suggesting their involvement in distinct steps of the GA biosynthetic pathway (Supplementary Fig. S3a). Additionally, we examined the co-expression correlations of these genes using high-resolution RNA sequencing data from developing vascular tissues, analyzed with RStudio software (https://rstudio.com/products/rstudio/download/). These analyses revealed that the GA biosynthetic genes clustered into two distinct groups, one of which contained all the highly expressed genes, suggesting that local GA biosynthesis may occur within the poplar stem (Supplementary Fig. S3b).

**Figure 2:**
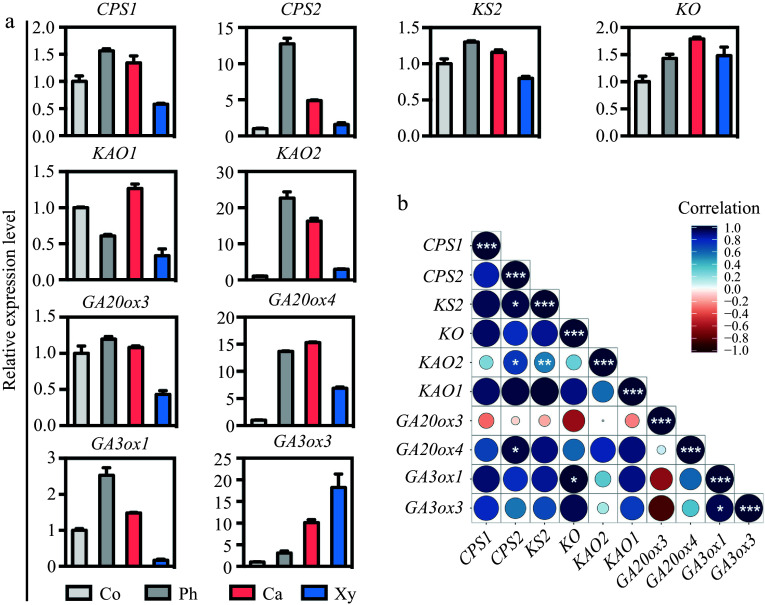
Expression patterns and correlation analysis of GA biosynthetic genes across wood-forming tissues in poplar stem. (a) The expression patterns of GA biosynthetic genes, including *CPS1/2*, *KS2*, *KO*, *KAO1/2*, *GA20ox3/4*, and *GA3ox1/3*, in various tissues of the poplar stem were assessed using micro-sections obtained through freezing microtome. The relative expression levels of each gene in the stem tissues were normalized to 1. Co = cortex; Ph = phloem; Ca = cambium; Xy = xylem. (b) Correlation analyses of the expression levels of GA biosynthetic genes in stem tissues were performed. Correlations were calculated based on a Student's t-distribution (degrees of freedom = n − 2, two-tailed test). Statistical significance is indicated as follows: *, *p* < 0.05; **, *p* < 0.01; ***, *p* < 0.001. Heatmaps were generated using RStudio software (R package: ggplot2). Positive correlations are represented by blue, while negative correlations are represented by red. The size of the circle corresponds to the strength of the co-expression correlation.

To validate the co-expression of GA biosynthetic genes in wood-forming tissues, we employed cryosectioning to collect cortex, phloem, cambium, and xylem tissues from poplar stems for RT-qPCR analysis. The accuracy of tissue collection was first confirmed using marker genes specific to each tissue type, including *ANT* for cambium^[26]^, *CLE41b* for phloem^[26]^, and *LMX5* for xylem^[15]^. RT-qPCR results confirmed that these marker genes were specifically expressed in their respective tissues (Supplementary Fig. S4), thereby verifying the precision of the cryosectioning method. Subsequent RT-qPCR analysis of GA biosynthetic genes revealed similar expression patterns in wood-forming tissues (Fig. 2a), consistent with the results from Supplementary Fig. S3. To further explore gene co-expression, we calculated the co-expression correlation coefficients and visualized the results in a heatmap using RStudio. The smaller the circular areas in the heat map, the higher the correlation between genes (Fig. 2b). These findings demonstrate a high level of correlation in the expression patterns of GA biosynthetic genes in poplar stems, supporting the hypothesis that local GA biosynthesis plays a critical role in regulating secondary growth.

### Local GA biosynthesis in poplar stems promotes wood formation

**Figure 3:**
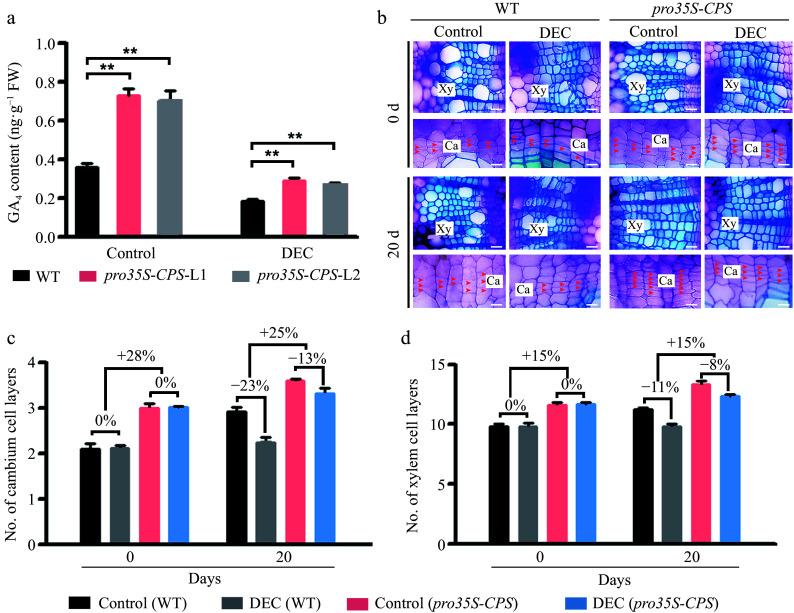
Overexpression of the *CPS* gene led to an increase of GA content in the stems of transgenic plants and the improvement of wood formation. (a) The GA_4_ content was measured in wild-type (WT) and two transgenic *pro35S-CPS* poplar lines using gas chromatography-mass spectrometry (GC-MS). Error bars represent ± standard deviation (SD). Asterisks indicate statistically significant differences between the control and decapitation treatment, as determined by one-way ANOVA followed by Dunnett's test for pairwise comparisons (**, *p* < 0.01; n = 3). (b) Cross-sections of WT and transgenic *pro35S-CPS* plants were analyzed 20 d after decapitation treatment. Red arrows indicate cambium cells. Scale bars = 50 μm. Ca, cambium; Xy, xylem; DEC, decapitation. (c) and (d) Quantification of cambium and xylem cell layers in poplar stems subjected to decapitation treatment. Error bars represent ± SD (n = 3).

Previous studies have demonstrated that CPS is a key rate-limiting enzyme in the bioactive gibberellin (GA) biosynthetic pathway in Arabidopsis^[34,35]^, rice^[36]^, and *Populus*^[25]^. Alignment of the amino acid sequences of AtCPS and PtrCPS paralogs revealed two conserved enzymatic domains, the N- and C-terpene synthase domains^[37]^ (Supplementary Fig. S5b). The three-dimensional structures of AtCPS, PtrCPS1, and PtrCPS2, as predicted by SWISS-MODEL (https://swissmodel.expasy.org) (Supplementary Fig. S5b), showed similar spatial arrangements, suggesting analogous enzymatic activities. To investigate whether locally synthesized GAs in the stem contribute to wood formation, *AtCPS* cDNA was driven by the constitutive *CaMV 35S* promoter (Fig. 3a). Over ten independent transgenic lines harboring *pro35S-CPS* were obtained (Supplementary Fig. S6b), and two lines with high *CPS* gene expression were selected (Supplementary Fig. S6b). Overexpression of *CPS* resulted in a 22%–25% increase in stem diameter and a 29%–34% increase in plant height (Supplementary Fig. S6c & S6d).

The bioactive GA (GA_4_) profile was determined in the developing stems of both *pro35S-CPS* and WT plants (Fig. 3b). As anticipated, GA_4_ levels were significantly elevated by 45%–50% in *pro35S-CPS* transformants compared to WT. To determine whether these changes in bioactive GA levels in poplar stems were due to local GA biosynthesis, GA_4_ concentrations were measured in decapitated and non-decapitated *pro35S-CPS* and WT plants. The results revealed that GA_4_ concentration was significantly higher in the wood-forming stems of transgenic plants overexpressing the *CPS* gene, regardless of decapitation treatment (Fig. 3a), indicating the presence of local GA biosynthesis in poplar stems.

To further assess the impact of local GA biosynthesis on vascular tissues, the *pro35S-CPS* lines were decapitated and stained with toluidine blue (Fig. 3b). Overexpression of *CPS* significantly enhanced cambium and xylem development compared to WT (Fig. 3b), although the effect on vascular development was reduced by decapitation (Fig. 3b). Quantitative analysis revealed a 13% and 23% reduction in the number of cambium cell layers in *pro35S-CPS* and WT plants, respectively, 20 days after decapitation (Fig. 3c). In contrast, the number of xylem cell layers was only slightly reduced in *pro35S-CPS* plants following decapitation compared to WT (Fig. 3d). These findings suggest that locally synthesized GAs play a role in regulating secondary vascular development in poplar stems.

### Local GA biosynthesis occurs in the cambium tissues of poplar stems

**Figure 4:**
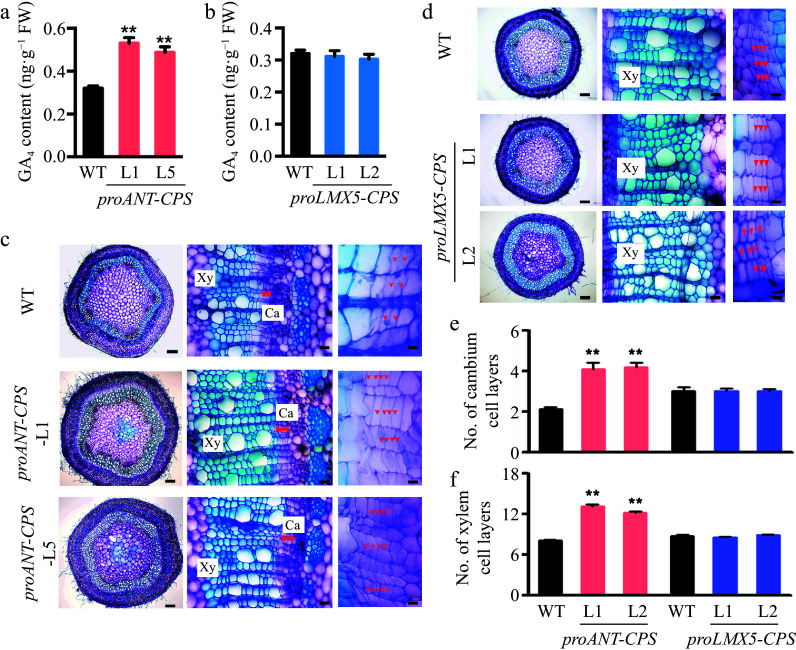
Cambium-specific expression of the *CPS* gene improved local GA synthesis in the stem of transgenic poplar plants and enhanced wood development. (a) Measurement of GA_4_ content in two transgenic *proANT-CPS* poplar lines by GC-MS. Error bars represent the ± standard deviation (SD). Asterisks denote statistically significant differences between the control and decapitation treatments (one-way ANOVA followed by Dunnett's test for pairwise comparisons; **, *p* < 0.01; n = 3). (b) Measurement of GA_4_ content in two transgenic *proLXM5-CPS* poplar lines by GC-MS. Error bars represent ± SD (n = 3). (c), (d) Cross-sections of WT, transgenic *proANT-CPS*, and *proLXM5-CPS* stems. Red arrows indicate cambium cells. Scale bars: left, 250 μm; middle and right, 50 μm. Ca: cambium; Xy: xylem. (e), (f) Quantification of cambium and xylem cell layers in poplar stems. Error bars represent ± SD. Asterisks indicate statistically significant differences between the control and decapitation treatments (one-way ANOVA followed by Dunnett's test for pairwise comparisons; **, *p* < 0.01; n = 3).

Given that GAs can be locally synthesized *de novo* in poplar stems, we aimed to identify the specific sites of GA biosynthesis that regulate wood development. To investigate this, we expressed the *CPS* genes in *Populus tomentosa* under the control of the cambium-specific *ANT* promoter^[26]^ and the xylem-specific *LMX5* promoter^[15]^. Several transgenic plants harboring the *proANT-CPS* and *proLMX5-CPS* constructs were generated, and two representative lines with high expression of each transgene were selected for further analysis (Supplementary Fig. S7). Notably, GA_4_ accumulation was significantly increased in the *proANT-CPS* transgenic plants compared to WT plants (Fig. 4a), whereas no significant changes in GA_4_ levels were observed in the *proLMX5-CPS* transgenic plants (Fig. 4b). Cross-sections of the 5^th^ internode revealed that overexpression of *CPS* in the cambium zone of *proANT-CPS* plants enhanced both cambium and xylem development (Fig. 4c). The number of cambium and xylem cell layers was significantly greater in the *proANT-CPS* lines than in WT plants (Fig. 4e & f). In contrast, no significant changes in cambium and xylem development were observed in the *proLMX5-CPS* lines (Fig. 4d−f).

**Figure 5:**
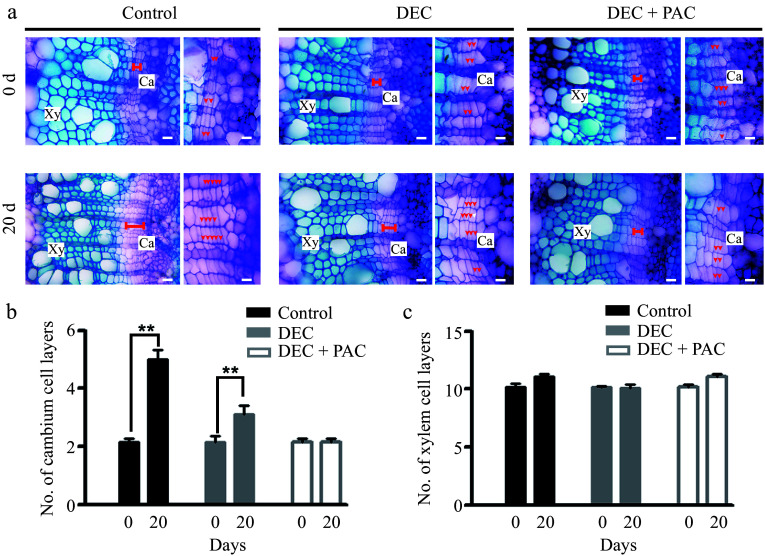
Locally biosynthesized GAs positively regulated cambial division in *proANT*-*CPS* transgenic poplar. (a) Cross-sectional images and toluidine blue staining of 4-week-old *proANT-CPS* transgenic lines subjected to decapitation and/or paclobutrazol (PAC) treatments. Transgenic plants were grown in WPM medium for 5 and 20 d under decapitation and 10 μM PAC treatments, respectively. Red dots indicate cambium cell layers. Scale bars = 50 μm. (b) Quantification of cambium cell layers in the stems of *proANT-CPS* transgenic lines after decapitation and/or PAC treatments. (c) Quantification of xylem cell layers in the stems of *proANT-CPS* transgenic lines following decapitation and/or PAC treatments. DEC, decapitation; PAC, 10 μM paclobutrazol. Error bars represent ± SD. Asterisks indicate significant differences in cambium cell layers after 20 d of decapitation treatment (Student's t-test; **, *p* < 0.01; n = 3).

To further elucidate the role of locally synthesized GAs in wood development, we decapitated the *proANT-CPS* lines to eliminate GA transport from the shoot apex and leaves (Fig. 5a). After 20 d of normal growth, the number of cambium cells in the *proANT-CPS* transgenic plants increased by 120%, whereas decapitation resulted in only a 40% increase in cambium cell number. Cambial cell development was completely inhibited in *proANT-CPS* plants treated with both paclobutrazol (PAC) and decapitation (Fig. 5b). Moreover, xylem development in these transgenic plants was not significantly affected by decapitation or PAC treatment (Fig. 5c). These results suggest that local GA biosynthesis in the vascular tissues of poplar stems positively regulates cambium cell division.

Previous studies have indicated that the first stage of the GA biosynthetic pathway occurs in chloroplasts^[1,2]^. Based on this, we hypothesized that, because xylem tissue lacks chloroplasts, it cannot provide the initial substrate for bioactive GA biosynthesis. To test this, we used confocal microscopy to observe chlorophyll autofluorescence in the stem, finding that the autofluorescence signal of chloroplasts was primarily localized to the bark and cambium tissues (Supplementary Fig. S8a). Quantitative analysis of the chloroplast autofluorescence distribution confirmed that chloroplasts are predominantly located in the cortex, phloem, and cambium zones (Supplementary Fig. S8b). These findings support the notion that local GA biosynthesis in poplar stems depends on the presence of chloroplasts.

### Locally biosynthesized GAs in stems regulate the expression of genes associated with cambial proliferation and differentiation

**Figure 6:**
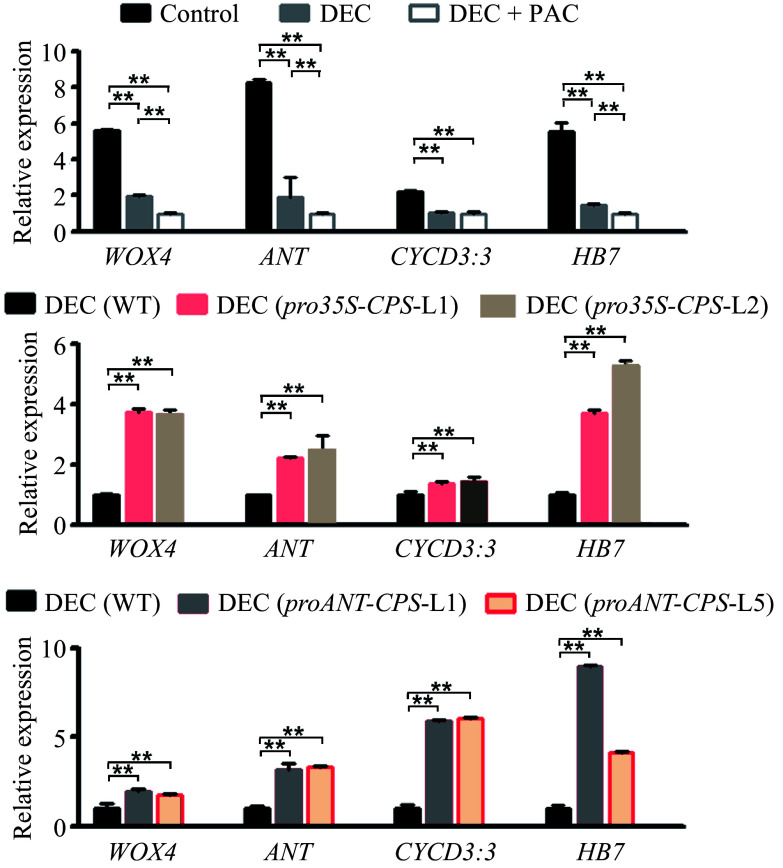
Expression levels of cambium development-related genes responding to local GA biosynthesis in poplar. (a) Relative expression levels of *WOX4*, *ANT*, *CYCD3:3*, and *HB7* in wild-type (WT) plants subjected to decapitation and/or PAC treatments. (b) Relative expression levels of *WOX4*, *ANT*, *CYCD3:3*, and *HB7* in WT and *pro35S:CPS* transgenic lines (L1 and L2) following 20 d of decapitation treatment. (c) Relative expression levels of *WOX4*, *ANT*, *CYCD3:3*, and *HB7* in WT and *proANT:CPS* transgenic lines after 20 d of decapitation treatment. Error bars represent the ± standard deviation (SD). Asterisks indicate statistically significant differences in gene expression levels between WT and each transgenic line, as determined by one-way ANOVA followed by Tukey's test for pairwise comparisons (**, *p* < 0.01; n = 3). DEC, decapitation; PAC, 10 μM paclobutrazol.

To investigate the effect of locally biosynthesized gibberellins (GAs) in poplar stems on secondary vascular development, we examined changes in the expression levels of genes related to vascular cell proliferation, including *WOX4*^[38]^, *ANT*^[39]^, *CYCD3*^[40,41]^, and *HB7*^[42]^, in WT and transgenic plants following decapitation and PAC treatments. The expression of these genes was significantly reduced in the stems of decapitated WT plants (Fig. 6a). Overexpression of the *CPS* gene resulted in a marked increase in the transcript accumulation of these marker genes in transgenic plants compared to WT, even under decapitation treatment (Fig. 6b). Similar results were observed in transgenic plants with *CPS* expression driven by the cambium-specific *ANT* gene promoter (Fig. 6c). These findings suggest that locally biosynthesized GAs in poplar stems play a crucial role in vascular development by regulating the expression of genes involved in cambium cell division.

## Discussion

Bioactive gibberellins (GAs) are well-established regulators of shoot growth and wood formation in woody species. A previous model of GA activity during wood development proposed that GA precursors are transported via the phloem from shoot leaves to distant sites^[25]^. This study aimed to investigate whether bioactive GAs are locally synthesized in poplar stems and to explore their role in wood formation. Previous studies have demonstrated that bioactive GAs accumulate in growing and elongating tissues, such as shoot apices, developing internodes, and expanding young leaves^[43,44]^, suggesting that GAs are predominantly synthesized at their site of action. In the present study, we identified *de novo* GA production in poplar stems following decapitation (Fig. 1a). GA synthesis was completely inhibited in decapitated stems treated with PAC (a GA biosynthesis inhibitor), resulting in a marked reduction in stem growth (Fig. 1b−e). These findings suggest that locally produced bioactive GAs in the stem play a crucial role in wood formation in poplar. Our results are consistent with previous studies in hybrid aspen (*Populus tremula × tremuloides*), where GAs induced secondary growth in decapitated stems even in the absence of auxin^[11]^. Additionally, we examined the expression of GA biosynthesis genes in poplar stems and found that at least one enzyme involved in each step of the GA biosynthetic pathway was highly expressed in the stem (Supplementary Fig. S2b). We also analyzed the co-expression of these genes in developing vascular tissues using RNA sequencing data (Supplementary Fig. S3b) and RT-qPCR (Fig. 2a), revealing strong correlations between different GA biosynthetic genes (Fig. 2b). These results indicate the presence of a complete GA biosynthetic pathway in poplar stems, highlighting the importance of local GA biosynthesis in regulating cambial activity and secondary growth.

In higher plants, *ent*-copalyl diphosphate synthase (CPS) encodes the first enzyme in the GA biosynthetic pathway^[25]^. Yamaguchi et al.^[18]^ demonstrated that the hydrophobic *ent*-kaurene can be intercellularly transported to produce bioactive GAs in Arabidopsis seeds. In aspen, *CPS* transcripts are detected in wood-forming tissues^[25]^, suggesting that *ent*-kaurene may be further metabolized to synthesize bioactive GAs in stems. In the current study, we examined transgenic plants carrying different constructs of the Arabidopsis *CPS* gene driven by either a constitutive *CaMV 35S* promoter or two vascular tissue-specific promoters (*ANT* and *LMX5*). Both promoters were expressed specifically in the stem: *ANT* is predominantly expressed in the vascular cambium^[26]^, while *LMX5* is highly expressed in the xylem^[15]^. Expression of *pro35S-CPS* in transgenic poplar plants led to elevated GA levels in the stem and promoted vascular tissue growth (Fig. 3). However, decapitation significantly reduced the increase in stem growth in these transgenic plants (Fig. 3), consistent with previous studies in poplar and tobacco (*Nicotiana tabacum*), where overexpression of *GA20ox* or *GID1* (a GA receptor) enhanced secondary stem growth^[5,15,45]^. In contrast, plants expressing *proANT-CPS* and *proLMX5-CPS* exhibited different levels of GA accumulation in the stem (Fig. 4a). Only the *CPS* gene driven by the *ANT* promoter increased GA levels and promoted secondary growth in the stems (Fig. 4a, c & d), whereas xylem-specific expression of *CPS* did not significantly affect GA biosynthesis or vascular growth (Fig. 4a, b & d). This suggests that the complete GA biosynthetic pathway may be absent in xylem tissues. However, transgenic poplar plants expressing *GA20ox1*, a key enzyme in GA biosynthesis, under the control of a developing xylem-specific promoter (*DX15*) exhibited accelerated stem growth and increased biomass^[46]^. Similarly, *pro35S-GA20ox* hybrid aspen plants exhibited significantly higher GA levels and enhanced xylem growth^[25,47]^, supporting the crucial role of GAs in regulating wood development.

Cambial cell differentiation in stems is regulated by both genetic and hormonal pathways^[8]^. Auxin is essential for cambial activity, and GAs act synergistically with auxin to promote cambial growth in stems^[11,48]^. In Arabidopsis, cambium cell division is controlled by the transcription factor WOX4, a key target of the CLAVATA3 (CLV3)/EMBRYO SURROUNDING REGION (ESR)-RELATED 41 (CLE41) signaling pathway^[38,49]^. Some *WOX* genes, specifically expressed in vascular tissues, have been shown to interact with the GA pathway to integrate the CLE/PXY pathway^[38,49]^. In this study, we investigated *WOX4* expression in WT and transgenic plants carrying *pro35S:CPS* and *proANT-CPS* constructs (Fig. 6). RT-PCR analysis revealed a significant increase in *WOX4* expression in the *pro35S:CPS* and *proANT-CPS* lines compared to WT plants (Fig. 6b & c). Interestingly, *WOX4* mRNA accumulation was markedly reduced in decapitated WT stems and further suppressed by PAC treatment. In contrast, *WOX4* expression in the *pro35S:CPS* and *proANT-CPS* lines remained high and unaffected by decapitation (Fig. 6b & c). Additionally, cytokinin has been shown to act synergistically with GAs and auxin to regulate secondary vascular development^[50,51]^. We analyzed the expression of *ANT* and *CYCD3:3*, the poplar orthologs of Arabidopsis *AINTEGUMENTA* (*ANT*) and *D-type cyclins* (*CYCD*), which are expressed in dividing cambial cells. Expression patterns similar to those of *WOX4* were observed in decapitated WT and transgenic plants expressing *pro35S:CPS* or *proANT-CPS* (Fig. 6). Overall, our findings suggest that locally synthesized GAs in stems can influence the expression of vascular formation-related genes and enhance cambium activity in poplar, providing new insights into the regulatory mechanisms of secondary growth in woody plants.

## SUPPLEMENTARY DATA

Supplementary data to this article can be found online.

## Data Availability

All data generated or analyzed during this study are included in this published article and its supplementary information files.
