# Supplementary material for: Locally biosynthesized gibberellins in Populus stems are involved in the regulation of wood development
Source: For Res (Fayettev). 2025 Feb 27;5:e005. doi: 10.48130/forres-0025-0005 (PMC11922183; doi:10.48130/forres-0025-0005)
Supplement: Supplementary file 1 — Supplementary data to this article can be found online. [file forres-0025-0005-Supplementary.zip › 10.48130_forres-0025-0005-Suppl-FigureS1.pdf]

Supplemental figure 1

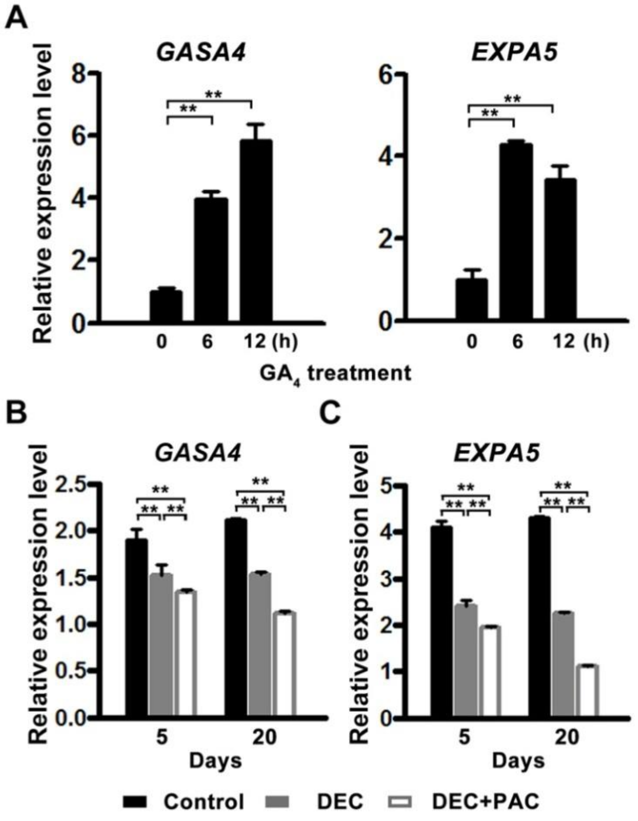

Figure S1. Expression analysis of GA-responsive genes in poplar stems following GA_4 treatment.

(A) Relative expression levels of *GASA4* and *EXPA5* under GA_4 treatment: Four-week-old poplar plants were cultivated in WPM medium supplemented with 50 μmol GA_4 for 6 or 12 hours. The stems were then collected for RNA extraction. The expression levels of these GA-responsive genes were measured using real-time RT-PCR. (B) Expression levels of *GASA4* and *EXPA5* under decapitation and PAC treatment: Poplar stems, cultivated in WPM medium for either 5 or 20 days, were collected for RNA extraction. The expression levels of bioactive GA-responsive genes were determined using real-time RT-PCR. Error bars represent ± SD, and asterisks indicate significant differences compared to wild-type (WT) values (one-way ANOVA followed by Tukey's test for pairwise comparisons): *, $P < 0.05$; **, $P < 0.01$.
