# Supplementary material for: Locally biosynthesized gibberellins in Populus stems are involved in the regulation of wood development
Source: For Res (Fayettev). 2025 Feb 27;5:e005. doi: 10.48130/forres-0025-0005 (PMC11922183; doi:10.48130/forres-0025-0005)
Supplement: Supplementary file 1 — Supplementary data to this article can be found online. [file forres-0025-0005-Supplementary.zip › 10.48130_forres-0025-0005-Suppl-FigureS2.pdf]

Supplemental figure 2

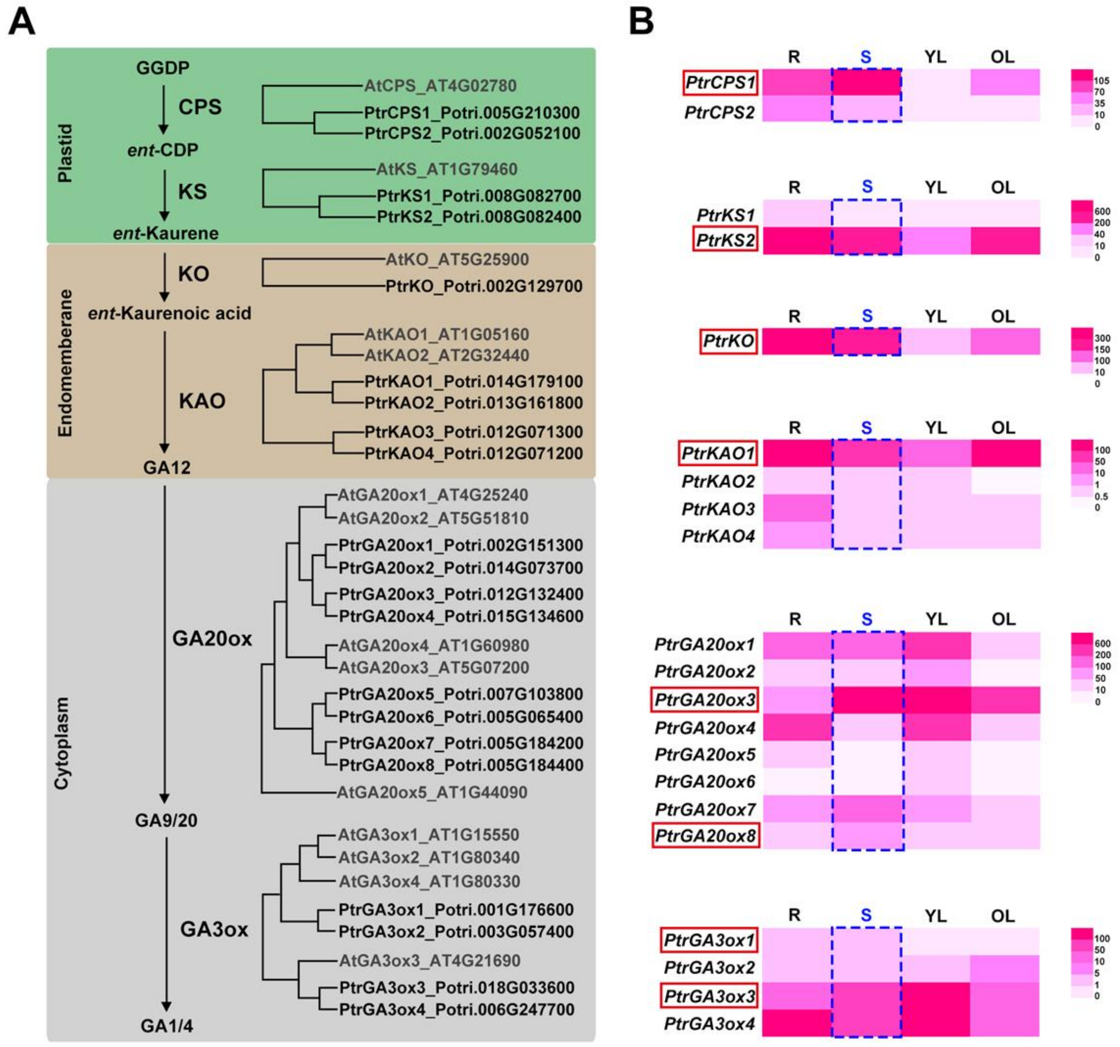

Figure S2. Expression levels of genes encoding GA biosynthetic enzymes in poplar.
(A) Phylogenetic relationship of gibberellin (GA) biosynthetic enzymes in *Arabidopsis* and their orthologs in *Populus trichocarpa*. Full-length amino acid sequences were retrieved from Phytozome (http://phytozome.jgi.doe.gov) and used for phylogenetic tree construction using MEGA6 software. Abbreviations: GGDP, geranylgeranyl pyrophosphate; *ent*-CDP, *ent*-copalyl pyrophosphate; CPS, *ent*-copalyl diphosphate synthase (*ent*-CDP); KS, *ent*-kaurene synthase; KO, *ent*-kaurene oxidase; KAO, *ent*-kaurenoic acid oxidase; GA20ox, GA20-oxidase; GA3ox, GA3-oxidase. (B) Transcript abundance of genes encoding GA biosynthetic enzymes across various *Populus* tissues, based on microarray datasets (http://bar.utoronto.ca/efppop/cgi-bin/efpWeb.cgi). The red frame highlights regions with relatively high expression in the stem. Tissue abbreviations: R, root; S, stem; YL, young leaves; OL, old leaves.
