# Supplementary material for: Locally biosynthesized gibberellins in Populus stems are involved in the regulation of wood development
Source: For Res (Fayettev). 2025 Feb 27;5:e005. doi: 10.48130/forres-0025-0005 (PMC11922183; doi:10.48130/forres-0025-0005)
Supplement: Supplementary file 1 — Supplementary data to this article can be found online. [file forres-0025-0005-Supplementary.zip › 10.48130_forres-0025-0005-Suppl-FigureS3.pdf]

Supplemental figure 3

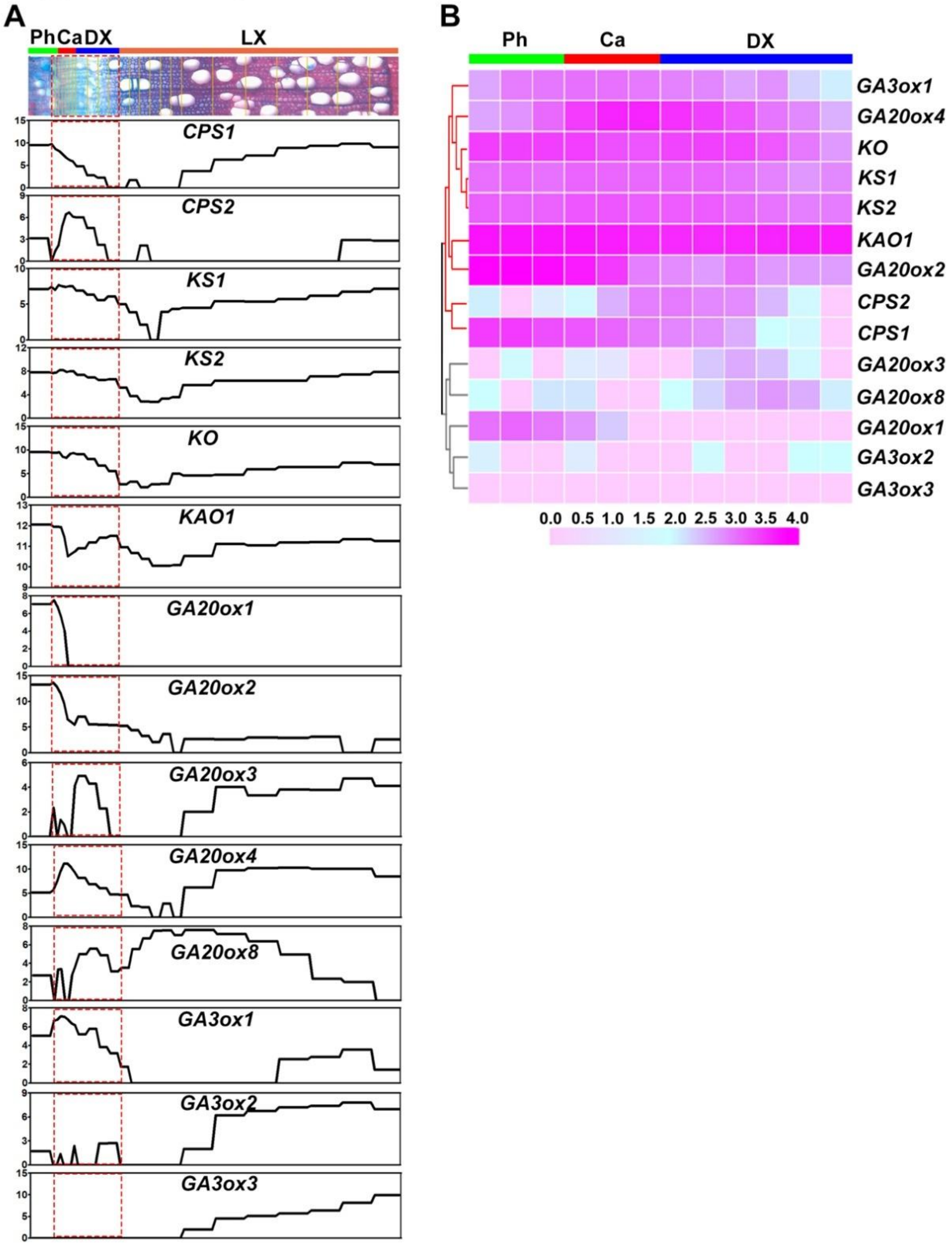

Figure S3. Expression patterns and co-expression correlations of genes encoding GA biosynthetic enzymes in wood-forming tissues of poplar stems.

(A) Expression patterns of genes encoding GA biosynthetic enzymes across the wood-forming tissues of poplar stems, based on the RNA-seq data from Tree 1 (Sundell et al., 2017). Curves were generated using expression values from cryo-sections of stem tissues. The red dotted lines represent early wood-forming tissues, including the phloem, cambium, and developing xylem. Abbreviations: Ph, phloem; Ca, cambium; DX, developing xylem; LX, lignified xylem. (B) Co-expression correlation of GA biosynthetic enzyme-encoding genes. Data is based on the RNA-seq databases corresponding to the red dotted lines in (A). The left-hand lines represent genes with co-expression correlations. Abbreviations: Ph, phloem; Ca, cambium; DX, developing xylem.
