# Supplementary material for: Locally biosynthesized gibberellins in Populus stems are involved in the regulation of wood development
Source: For Res (Fayettev). 2025 Feb 27;5:e005. doi: 10.48130/forres-0025-0005 (PMC11922183; doi:10.48130/forres-0025-0005)
Supplement: Supplementary file 1 — Supplementary data to this article can be found online. [file forres-0025-0005-Supplementary.zip › 10.48130_forres-0025-0005-Suppl-FigureS4.pdf]

Supplemental figure 4

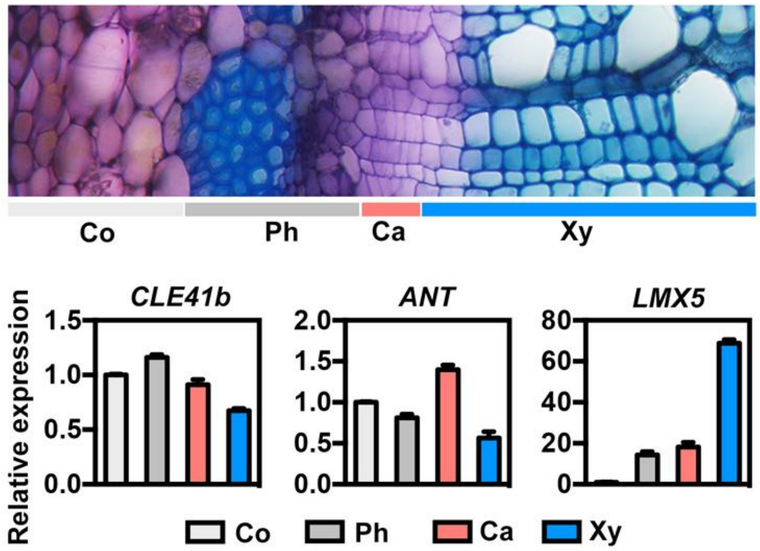

Figure S4. Expression profiles of selected vascular-specific genes in poplar stems.
The three-month-old poplar plants grown in a greenhouse were microdissected into four distinct cryo-fractions: cortex, phloem, cambium, and xylem, using a frozen slicer. RNA was then extracted from these fractions for subsequent analysis. The expression levels of vascular-specific genes were assessed using real-time RT-PCR. *CLE41b* was specifically expressed in the phloem, ANT in the cambium, and *LMX5* in the xylem. Abbreviations used: Co, cortex; Ph, phloem; Ca, cambium; Xy, xylem.
