# Supplementary material for: Locally biosynthesized gibberellins in Populus stems are involved in the regulation of wood development
Source: For Res (Fayettev). 2025 Feb 27;5:e005. doi: 10.48130/forres-0025-0005 (PMC11922183; doi:10.48130/forres-0025-0005)
Supplement: Supplementary file 1 — Supplementary data to this article can be found online. [file forres-0025-0005-Supplementary.zip › 10.48130_forres-0025-0005-Suppl-FigureS5.pdf]

Supplemental figure 5

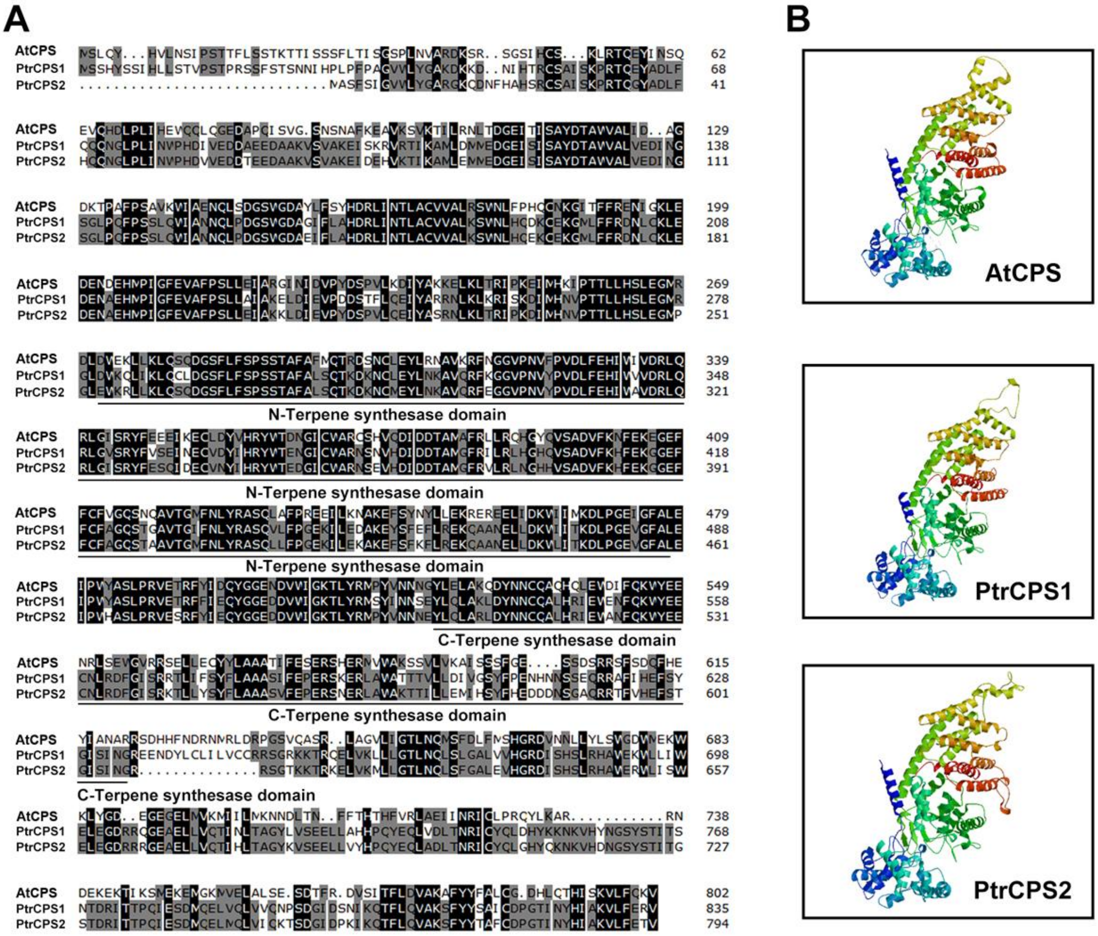

Figure S5. Sequence and protein structure analysis of Arabidopsis CPS and *Populus trichocarpa* CPS1/2.

(A) The amino acid sequences of these proteins were aligned using DANMAN8. Black shading indicates conserved amino acid sequences, while grey shading denotes similar amino acids. The PtrCPS1/2 protein contains conserved N-terpene synthase and C-terpene synthase domains, which are underlined, and these are aligned with the corresponding domains in the Arabidopsis CPS protein. Identical and similar amino acids are highlighted in black and grey, respectively. (B) Protein structure analysis of Arabidopsis CPS and *Populus trichocarpa* CPS1/2 was conducted using the SWISS-MODEL platform (https://swissmodel.expasy.org/assess/6v7DcH/01). The blue color highlights the two conserved domains of the CPS enzyme.
