# Supplementary material for: Locally biosynthesized gibberellins in Populus stems are involved in the regulation of wood development
Source: For Res (Fayettev). 2025 Feb 27;5:e005. doi: 10.48130/forres-0025-0005 (PMC11922183; doi:10.48130/forres-0025-0005)
Supplement: Supplementary file 1 — Supplementary data to this article can be found online. [file forres-0025-0005-Supplementary.zip › 10.48130_forres-0025-0005-Suppl-FigureS6.pdf]

Supplemental figure 6

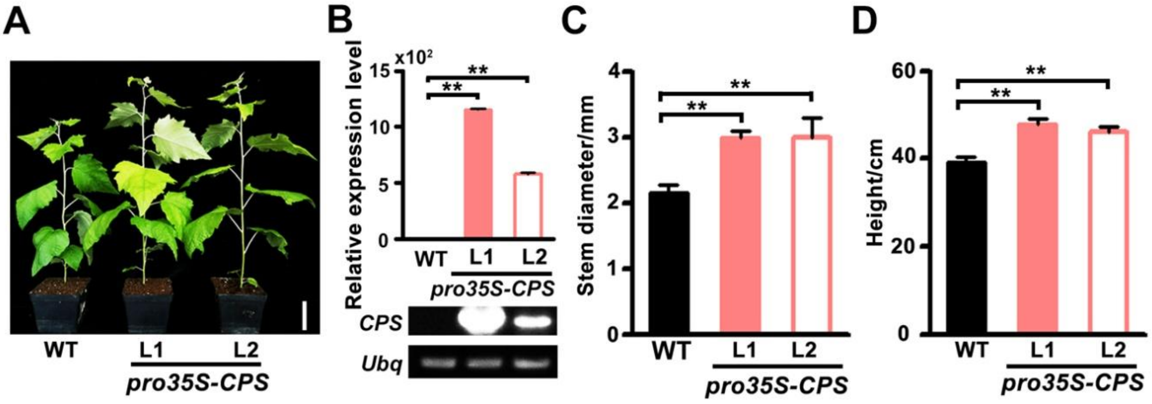

Figure S6. Phenotypic analysis and expression levels of *CPS* in transgenic poplar plants.
(A) Phenotype of *pro35S-CPS* transgenic poplar lines grown in a greenhouse. Scale bar = 5 cm. **(B)** Expression analysis of the *CPS* gene in two independent transgenic and wild-type plants, grown in the greenhouse for 3 months, using quantitative and semi-quantitative RT-PCR, respectively. RNA was extracted from the 3rd to 8th internodes of both WT and transgenic plants. Ubiquitin was used as the reference gene. Error bars represent ± SD. Asterisks denote significant differences relative to the WT values (one-way ANOVA followed by Dunnett's test for pairwise comparisons): *, $P < 0.05$; **, $P < 0.01$. **(C)** and **(D)** Measurement of stem diameter (C) and plant height (D) in two independent *pro35S-CPS* lines (L1 and L2).
