# Supplementary material for: Locally biosynthesized gibberellins in Populus stems are involved in the regulation of wood development
Source: For Res (Fayettev). 2025 Feb 27;5:e005. doi: 10.48130/forres-0025-0005 (PMC11922183; doi:10.48130/forres-0025-0005)
Supplement: Supplementary file 1 — Supplementary data to this article can be found online. [file forres-0025-0005-Supplementary.zip › 10.48130_forres-0025-0005-Suppl-FigureS7.pdf]

Supplemental figure 7

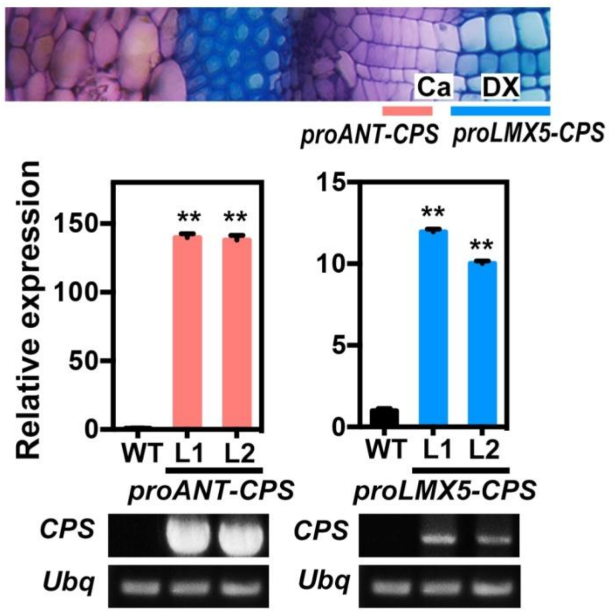

Figure S7. Expression analysis of the *CPS* gene in transgenic plants harboring *proANT-CPS* and *proLMX5-CPS* constructs.

Transgenic plants were grown in a greenhouse for three months. RNA was extracted from the third to eighth internodes of both wild-type (WT) and transgenic plants for quantitative and semi-quantitative RT-PCR analysis. The ubiquitin gene served as the reference gene. Error bars represent the standard deviation (± SD). Asterisks indicate statistically significant differences compared to the WT values, as determined by one-way ANOVA followed by Dunnett's test for pairwise comparisons: *, $P < 0.05$; **, $P < 0.01$.
