# Supplementary material for: Locally biosynthesized gibberellins in Populus stems are involved in the regulation of wood development
Source: For Res (Fayettev). 2025 Feb 27;5:e005. doi: 10.48130/forres-0025-0005 (PMC11922183; doi:10.48130/forres-0025-0005)
Supplement: Supplementary file 1 — Supplementary data to this article can be found online. [file forres-0025-0005-Supplementary.zip › 10.48130_forres-0025-0005-Suppl-FigureS8.pdf]

Supplemental figure 8

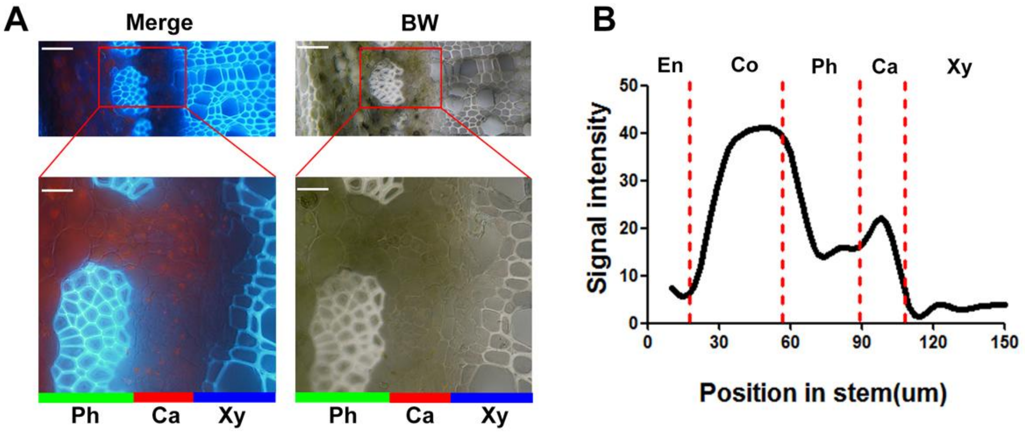

Figure S8. Chloroplast distribution in poplar stems.

(A) Chloroplast autofluorescence activity in the 7th internode stem of 3-month-old poplar plants. Red indicates chloroplast autofluorescence, while blue represents lignin autofluorescence. BW: Bright White; Ph: Phloem; Ca: Cambium; Xy: Xylem. Scale bars: upper = 100 μm, lower = 50 μm. (B) Quantification of the average chloroplast autofluorescence activity distribution in the poplar stem. The red dotted lines demarcate the boundaries of different stem tissues. En: Endoepidermis; Ph: Phloem; Ca: Cambium; Xy: Xylem.
