# Supplementary material for: Locally biosynthesized gibberellins in Populus stems are involved in the regulation of wood development
Source: For Res (Fayettev). 2025 Feb 27;5:e005. doi: 10.48130/forres-0025-0005 (PMC11922183; doi:10.48130/forres-0025-0005)
Supplement: Supplementary file 1 — Supplementary data to this article can be found online. [file forres-0025-0005-Supplementary.zip › 10.48130_forres-0025-0005-Suppl-TableS1.pdf]

Table S1. Sequences of oligonucleotide primers used in this study

Name	Sequence
**For plasmid construction**	
AtCPS-CDS-fw	5'-ATGTCTCTTCAGTATCATGT-3'
AtCPS-CDS-rv	5'-CTAGACTTTTTGAAACAAGA-3'
AtCPS-CDS-*Bam*HI-fw	5'-CGGGATCCATGTCTCTTCAGTATCATGT -3'
AtCPS-CDS-*Mlu*I-fw	5'-CGACGCGTCTAGACTTTTTGAAACAAGA -3'
Pro-PtoANT-fw	5'- AGTATTATGTTCCAAGCT -3'
Pro-PtoANT-rv	5'- CTTCTCCTCCTCTCACTC -3'
*Pro-PtoANT-Eco*RI-fw	5'- CGGAATTCAGTATTATGTTCCAAGCT -3'
*Pro-PtoANT-Bam*HI-rv	5'- CGGGATCCCTTCTCCTCCTCTCACTC -3'
Pro-PtoLMX5-fw	5'- AATGTGGGCCTGGTGTTATA -3'
Pro-PtoLMX5-rv	5'- GGTTGGTGGGGAAAGATG -3'
*Pro-PtoLMX5-Eco*RI-fw	5'-GGAATTCAATGTGGGCCTGGTGTTATA -3'
*Pro-PtoLMX5-Bam*HI-rv	5'-CGGGATCCGGTTGGTGGGGAAAGATG -3'
**For PCR genotyping**	
Hygromycin-fw	5’ ATCGGACGATTGCGTCGCATC 3’
Hygromycin-rv	5'-GTGTCACGTTGCAAGACCTG-3'
Kana-fw	5’ GTCGACATGGATGGATTGCACG 3’
Kana-rv	5’ GTCGACTCAGAAGAACTCGTCAAGAAG 3’
**For qRT-PCR**	
PtrANT-q-fw	5'-AGTCCTGGTTCGCAGTCAA-3'
PtrANT-q-rv	5'-CAGTCCATCTGTGCCTTGTAA-3'
*PtrWOX*4-q-fw	5'-GAGAACTCCTAATGGGCAACA-3'
PtrWOX4ab-q-rv	5'-ATAATGGTAATGGGAGATGGG-3'
PtrCYCD3:3-q-fw	5'-GGTCTTTGCGGACATCGT-3'
PtrCYCD3:3-q-rv	5'-TGGTGGGGAACTTTGGAA-3'
PtrEXPA1-q-fw	5'-GTAACAGCATTGTTGATTGGGT-3'
PtrEXPA1-q-rv	5'-GGTGCATAGCAAAGCAATTCTA-3'
PtrGASA4-q-fw	5'-GGTGAGACTATATTATGGGGCC-3'
PtrGASA4-q-rv	5'-GTCTTCCAGTTGTTGTAGCAAG-3'
PtrHB7-q-fw	5'-GCAGAAATGTTGCCTAGCGG-3'
PtrHB7-q-rv	5'-GCCGCCATTGTTGTCTTCTG-3'
